# Data on the fluorescence quenching analysis of BSA induced by pyrene and/or 1-hydroxypyrene in binary and ternary systems

**DOI:** 10.1016/j.dib.2018.08.140

**Published:** 2018-08-30

**Authors:** Jing Zhang, Linfeng Chen, Dan Liu, Yaxian Zhu, Yong Zhang

**Affiliations:** a Key Laboratory of Estuarine Ecological Security and Environmental Health, Tan Kah Kee College, Xiamen University, Zhangzhou 363105, PR China; b State Key Laboratory of Marine Environmental Sciences of China (Xiamen University), College of Environment and Ecology, Xiamen University, Xiamen 361102, PR China; c Department of Chemistry, College of Chemistry and Chemical Engineering, Xiamen University, Xiamen 361005, PR China

## Abstract

This article present data related to the publication entitled “Interactions of pyrene and/or 1-hydroxypyrene with bovine serum albumin based on EEM-PARAFAC combined with molecular docking” (Zhang et al., 2018) [1]. The excitation-emission matrix (EEM) fluorescence spectral parameters of pyrene, 1-hydroxypyrene, bovine serum albumin (BSA), and their mixtures were presented in this article. Combined EEM - parallel factor analysis with fluorescence quenching analysis, some data related to the binding affinity of pyrene and/or 1-hydroxypyrene with BSA in the binary and ternary systems were obtained.

**Specifications Table**

**Table:** 

Subject area	*Chemistry*
More specific subject area	*Analytical chemistry*
Type of data	*Table, figure*
How data was acquired	*FLS 920 steady/transient fluorescence spectrometer (Edinburgh, UK)*
Data format	*Raw, analyzed*
Experimental factors	*For all of the excitation-emission matrix (EEM) spectroscopy measurements, different concentrations of Pyrene, 1-hydroxypyrene, and BSA were prepared in 0.05 mol L*^*−1*^*Tris–HCl buffer (pH = 7.40, containing 0.10 mol L*^*−1*^*NaCl), with each containing ethanol of no more than 0.5‰.*
Experimental features	*Combined with EEM fluorescence spectra with parallel factor analysis to investigate the binding ability of BSA with pyrene and/or 1-hydroxypyrene*
Data source location	*State Key Laboratory of Marine Environmental Sciences of China (Xiamen University) (MEL), Xiamen university, Xiamen, China*
Data accessibility	*Data are provided with this article*
Related research article	*Interactions of pyrene and/or 1-hydroxypyrene with bovine serum albumin based on EEM-PARAFAC combined with molecular docking*[1]

**Value of the data**

● The EEM data showed that the spectra of pyrene, 1-hydroxypyrene and BSA overlap, and this work provided the way to solve it, which may provide an insight to study other similar ternary interaction systems.
● The data showed the difference of the binding affinity of pyrene or 1-hydroxypyrene with BSA both in the binary and ternary systems, which was helpful for readers to compare the binding affinity of pyrene or 1-hydroxypyrene with BSA.
● The data may be further helpful for understanding the combined toxicity of pyrene and 1-hydroxypyrene.

## 1 Data

### 1.1 Excitation-emission matrix (EEM) fluorescence spectral parameters of Pyrene (Pyr), 1-hydroxypyrene (1-OHPyr), bovine serum albumin (BSA), and their mixtures

Table 1 showed the EEM fluorescence spectral parameters of Pyr (6.0 × 10^−7^ mol L^−1^), 1-OHPyr (7.5 × 10^−7^ mol L^−1^), BSA (1.0 × 10^−6^ mol L^−1^), and their mixtures. The range of excitation/emission wavelengths of Pyr, 1-OHPyr, BSA, and their mixtures, and the maximum excitation/emission wavelength of each were presented. As can be seen, the excitation spectra of Pyr were in the range of 260–278 and 295–340 nm, with the emission spectra ranging from 370 to 440 nm; for 1-OHPyr, its excitation spectra ranged from 260 to 290 nm and 310 to 370 nm, with the emission spectra ranging from 380 to 440 nm; however, excitation spectra of BSA ranged from 250 to 310 nm, with the emission spectra in the range of 290–450 nm. The data indicated that the EEM spectra of the mixed system of BSA, Pyr, and/or 1-OHPyr overlap severely.

**Table 1:** Fluorescence spectra properties of Pyr, 1-OHPyr, BSA, and their mixtures.^a^

System	*λ_ex_* (nm)	*λ*_em_ (nm)	Peak position *λ_ex_* (nm)	Peak position *λ_em_* (nm)
Pyr	260–278,	370–440	333	372
	295–340			
1-OHPyr	260–290,	380–440	346	386
	310–370			
BSA	250–310	290–450	280	341
Mixtures	250–390	290–460	335	388

a *C*_BSA_ = 1.0 × 10^−6^ mol L^−1^; *C*_1-OHPyr_ = 7.5 × 10^−7^ mol L^−1^; *C*_Pyr_ = 6.0 × 10^−7^ mol L^−1^

### 1.2 Fluorescence quenching data of BSA by Pyr and 1-OHPyr in the binary and ternary systems using Stern–Volmer plots

**Fig. 1:**
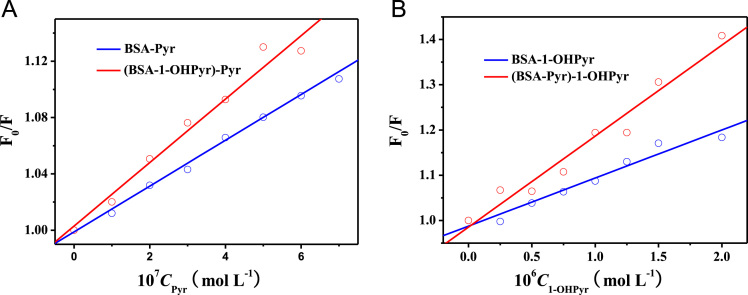
Stern–Volmer plots for the quenching of BSA by Pyr and 1-OHPyr in the binary and ternary systems.

After using the parallel factor analysis to decompose the EEM spectra of complex systems of Pyr, 1-OHP and BSA, the fluorescence properties of each individual component were obtained [1]. Combined with the fluorescence quenching method, the interactions of BSA with Pyr and 1-OHPyr in binary and ternary interaction systems were further studied. The measured fluorescence quenching data of BSA by Pyr and 1-OHPyr in the binary and ternary systems were fitted by Origin 7.5 based on Stern–Volmer plots (Eq. (1)) (Fig. 1) [2], and corresponding values were listed in Table 2.

(1) $$F_{0}/F=1+K_{sv}[Q]=1+K_{q}\tau_{0}[Q]$$

**Table 2:** *K_sv_* and *K_q_* values for Pyr - BSA and 1-OHPyr-BSA in the binary and ternary systems.

System	10^−5^*K_sv_* (L mol^−1^)	10^–13^*K_q_* (L mol^−1^ s^−1^)	*R*^a^
BSA-Pyr	1.63	1.63	0.992
(BSA-1-OHPyr)-Pyr	2.25	2.25	0.990
BSA-1-OHPyr	1.06	1.06	0.980
(BSA-Pyr)-1-OHPyr	2.01	2.01	0.980

a *R* is the correlation coefficient.

*F*_*0*_ and *F* are the relative fluorescence intensities of BSA in the absence and presence of Pyr or 1-OHPyr, respectively; [*Q*] is the concentration of Pyr or 1-OHPyr, *K*_*sv*_ is the Stern–Volmer quenching constant, and *K*_*q*_ is the quenching rate constant.

**Fig. 2:**
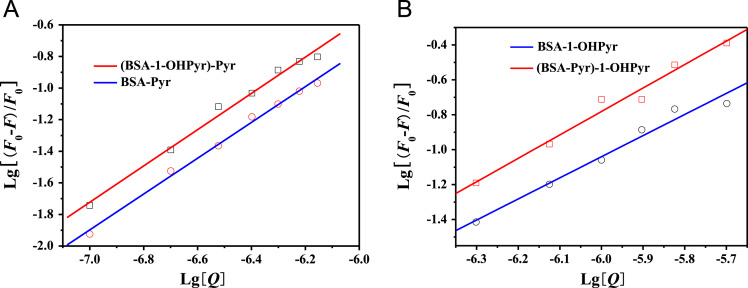
Double logarithm plots for the quenching of BSA by Pyr and 1-OHPyr in binary and ternary systems.

Fig. 2 showed the double logarithm plots for the quenching of BSA by Pyr and 1-OHPyr in binary and ternary systems. The binding constant (*K*_**b**_) and the number of binding sites (*n*) of Pyr and 1-OHPyr with BSA in binary and ternary interaction systems could be calculated by fitting the curve based on Eq. (2) [3], [4].

(2) $$Lg\frac{F_{0}-F}{F}=LgK_{b}+nLg[Q]$$

## 2 Experimental design, materials, and methods

### 2.1 Materials

BSA (purity > 99.5%), Pyr (purity > 98%) and 1-OHP (purity > 99%) were purchased from Sigma Chemical Company (St. Louis, MO, USA) and were used without further purification. Stock solutions of 4.0 × 10^−5^ mol L^−1^ BSA were prepared in 0.05 mol L^−1^ Tris–HCl buffer (pH = 7.40, containing 0.10 mol L^−1^ NaCl). Stock solutions of 1-OHP and Pyr were prepared individually in ethanol at concentrations of 2.0 × 10^−3^ mol L^−1^. The stock solutions were stored at 277 K in the dark. All of the other chemicals that were used were of analytical reagent grade. Milli-Q water (18.2 MΩ cm) was used throughout the study.

### 2.2 Methods

#### 2.2.1 EEM fluorescence spectra of Pyr, 1-OHPyr, BSA, and their mixtures

For all of the EEM spectroscopy measurements, 32 samples of different concentrations of Pyr, 1-OHPyr, and BSA were prepared with Tris–HCl buffer (Table 3), with each containing ethanol of no more than 0.5‰. Moreover, pure solutions of Pyr (6.0 × 10^−5^ mol L^−1^), 1-OHPyr (7.5 × 10^−5^ mol L^−1^), and BSA (1.0 × 10^−6^ mol L^−1^) were also prepared in Tris–HCl buffer as reference samples. After equilibration at 25 ± 1 °C for 20 min, EEM measurements were carried out on an FLS 920 steady/transient fluorescence spectrometer (Edinburgh, UK), which was equipped with a 150-W xenon lamp. Samples were measured in the excitation range of 250–410 nm (every 5 nm) and emission range of 280–500 nm (every 2 nm) using a 10-mm quartz cuvette. The excitation and emission slits were both set at 1 nm. Thus, the EEM dataset had the dimensions of 33 (Ex.) × 111 (Em.) × 35 (Samples).The data showed in Section 1.1 was the EEM fluorescence spectra of pure solutions of Pyr (6.0 × 10^−5^ mol L^−1^), 1-OHPyr (7.5 × 10^−5^ mol L^−1^), BSA (1.0 × 10^−6^ mol L^−1^) and their mixed solution (sample 7: *C*_BSA_ = 1.0 × 10^−6^ mol L^−1^, *C*_1-OHPyr_ = 7.5 × 10^−7^ mol L^−1^, *C*_Pyr_ = 6.0 × 10^−7^ mol L^−1^).

**Table 3:** The concentration setups of Pyr, 1-OHPyr, and BSA in 32 samples [1].

Nos.	10^6^ Pyr (mol L^−1^)	10^7^ 1-OHPyr (mol L^−1^)	10^6^ BSA (mol L^−1^)	Nos.	10^6^ Pyr (mol L^−1^)	10^7^ 1-OHPyr (mol L^−1^)	10^6^ BSA (mol L^−1^)
**1**	0	7.5	1.0	**17**	0	0	1.0
**2**	0.1	7.5	1.0	**18**	0.1	0	1.0
**3**	0.2	7.5	1.0	**19**	0.2	0	1.0
**4**	0.3	7.5	1.0	**20**	0.3	0	1.0
**5**	0.4	7.5	1.0	**21**	0.4	0	1.0
**6**	0.5	7.5	1.0	**22**	0.5	0	1.0
**7**	0.6	7.5	1.0	**23**	0.6	0	1.0
**8**	0.7	7.5	1.0	**24**	0.7	0	1.0
**9**	0.6	0	1.0	**25**	0	0	1.0
**10**	0.6	2.5	1.0	**26**	0	2.5	1.0
**11**	0.6	5.0	1.0	**27**	0	5.0	1.0
**12**	0.6	7.5	1.0	**28**	0	7.5	1.0
**13**	0.6	10.0	1.0	**29**	0	10.0	1.0
**14**	0.6	12.5	1.0	**30**	0	12.5	1.0
**15**	0.6	15.0	1.0	**31**	0	15.0	1.0
**16**	0.6	20.0	1.0	**32**	0	20.0	1.0

Samples 1–8: mixed BSA with 1-OHPyr first, then addition of Pyr; Samples: 9–16: mixed BSA with Pyr first, then addition of 1-OHPyr.

#### 2.2.2 PARAFAC analysis

Using MATLAB R2016b software (the MathWorks, Inc., Natick, MA, USA), the EEM dataset (size of 33 × 111 × 35) was decomposed by the PARAFAC method, according to the procedures described by Ref. [5]. First, the EEM spectra of the samples were corrected by subtracting the EEM spectra of the blank samples (Tris–HCl buffer) and removing the Rayleigh scattering and the two order Raman scattering peaks to reduce the scattering light and other background interference. Second, the 2–8 component model is used to decompose the EEM dataset by trilinear decomposition. To reduce the time of Matlab analysis, the relative fluorescence intensity values of the 35 EEM spectra were reduced by 100 times; thus, the relative fluorescence intensity values of the three components reported in this section were 0.01 times their practically measured values. Finally, the relative fluorescence intensity values of BSA obtained were used for the fluorescence quenching analysis.
