# Supplementary material for: Data on the fluorescence quenching analysis of BSA induced by pyrene and/or 1-hydroxypyrene in binary and ternary systems
Source: Data Brief. 2018 Aug 30;20:927–32. doi: 10.1016/j.dib.2018.08.140 (PMC6138979; doi:10.1016/j.dib.2018.08.140)
Supplement: Supplementary file 1 — Supplementary material [file mmc1.docx]

Conflict of Interest

The authors declare that there is no conflict of interest.
